# Bipolar head dissociation: radiographic assessment of bipolar hemiarthroplasty dislocation and the role of the “O” sign

**DOI:** 10.1007/s00256-026-05233-z

**Published:** 2026-04-25

**Authors:** Elliot F. Forst, Sami Alaraj, William M. Weiss

**Affiliations:** 1 John Sealy School of Medicine, University of Texas Medical Branch, Galveston, TX USA; 2 Department of Orthopedic Surgery, University of Texas Medical Branch, Galveston, TX USA

**Keywords:** Bipolar, Dislocation, Dissociation, Hemiarthroplasty, O sign, Reduction

## Abstract

Bipolar hemiarthroplasty dislocation is an uncommon but clinically significant complication of closed reduction that often necessitates revision, causing prolonged recovery, increased morbidity, and higher costs. A recently described radiographic marker, the “O sign”—a concentric circle appearance of the prosthesis on radiographs—has been proposed as an indicator that closed reduction may be safely attempted. While potentially useful for pre-reduction assessment, its predictive value remains uncertain. We report a case evaluating the diagnostic value of the O sign in bipolar hemiarthroplasty dislocation. A patient underwent both radiography and computed tomography (CT) prior to closed reduction. Radiographic features, including concentricity of the external shell and central positioning of the inner femoral head, were assessed for alignment and eccentricity. On anteroposterior radiographs, the external shell displayed a concentric “O” appearance. However, closer inspection revealed unequal circumferential shell thickness suggesting anterior rotation. CT imaging confirmed eccentric positioning of the femoral head within the outer shell, consistent with early bipolar head dissociation. This case demonstrates that while a concentric O sign on radiographs may suggest closed reduction is feasible, subtle misalignments such as asymmetric shell thickness or eccentric head positioning can be missed without CT. The predictive value of the O sign therefore depends not only on external shell symmetry but also on internal head alignment. Failure to meet either criterion may indicate early dissociation and warrants caution before attempting closed reduction. By integrating CT into assessment, the O sign may evolve from a binary marker to a more refined tool for guiding management of bipolar hemiarthroplasty dislocations.

## Introduction

Hemiarthroplasty is the most widely used operative treatment for displaced femoral neck fractures in older adults [1]. Although widely regarded as the standard of care, one rare yet important complication—bipolar head dissociation—has been documented following closed reduction attempts for dislocated hip hemiarthroplasties [2]. Institutional series estimate dissociation on the order of approximately 1% of hemiarthroplasties, ultimately requiring operative management [2].

A plausible mechanism for these dissociations is the “bottle opener effect,” wherein the outer shell catches on the acetabular rim during traction and is levered off the inner head, producing intraprosthetic disassembly [3]. Because the repeated or forceful maneuvers associated with a closed reduction attempt can lead to this “bottle-opener effect,” early transition to open reduction/revision is often advised [2]. In 2023, Sim et al*.* described the “O sign”—a radiographic finding characterized by concentric circles of the bipolar prosthesis seen on anteroposterior hip or pelvis views of a dislocated hemiarthroplasty—as a predictor that closed reduction may be safely attempted [4]. As a newly described finding, the O sign warrants further investigation to determine its reliability, but it may ultimately serve as a useful decision-making tool in the management of dislocated hemiarthroplasties.

This case report examines the O sign by correlating anteroposterior radiographs with computed tomography in a patient who developed bipolar head dissociation following hemiarthroplasty, with the goal of refining practical recognition and management considerations grounded in current literature. Written informed consent for a case report was obtained from the patient.

## Case report

An 87-year-old white male was presented to the emergency department by EMS for a complaint of right hip pain after falling from a bar stool. Workup with imaging demonstrated a displaced right femoral neck fracture. Medical history is significant for limited baseline mobility and multiple comorbidities including chronic kidney disease, atrial fibrillation on anticoagulation, prior stroke, and a recent hospitalization for congestive heart failure. The patient underwent a cemented right hip hemiarthroplasty (HHA). A standard, posterior approach was utilized. The femoral canal was prepared and broached to size 7, and a cemented femoral stem with a 127° neck angle was implanted (Accolade Universal OD12 mm, Stryker). A C-Taper LFIT +7.5 mm offset head was used in combination with a 28 mm × 54 mm UHR bipolar head. Simplex bone cement was utilized to secure both the femoral stem and spacer components. Trial reductions demonstrated appropriate leg length and excellent joint stability prior to final implantation.

**Fig. 1:**
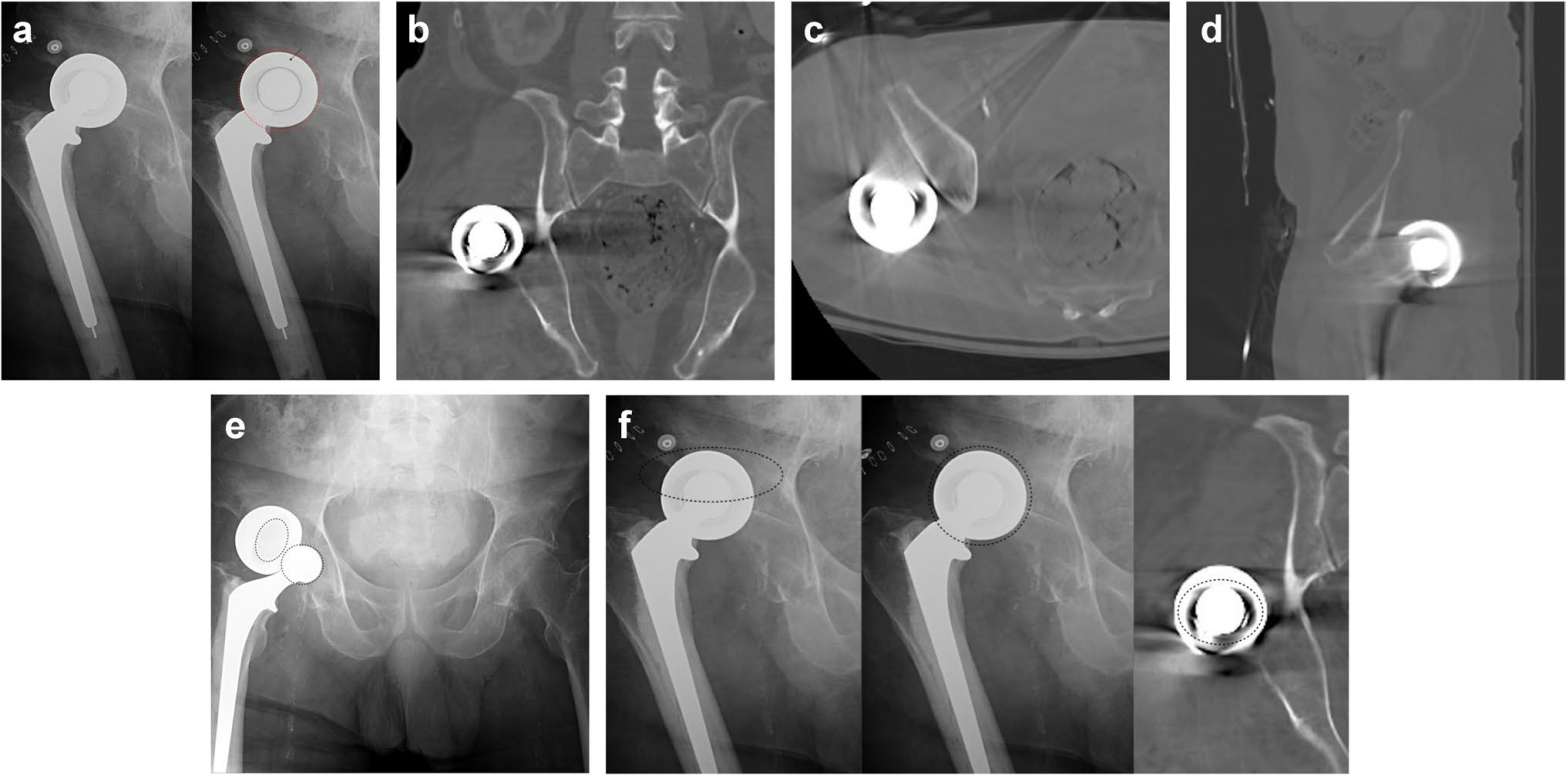
**a** Anterior/posterior radiograph of the pelvis demonstrating dislocation of right hemiarthroplasty prior to any attempts at reduction. Left: unannotated image. Right: annotated overlay highlighting the “O”-shaped borders of the outer bipolar shell (red ring) and inner femoral head (black ring). Arrow denotes asymmetric radiodensity from anterior rotation of the outer shell. **b** Coronal CT with contrast shows the outer shell of the bipolar head anteriorly rotated. A concentric O-shaped radiodense rim with asymmetric wall thickness is seen, with the eccentrically positioned femoral head producing an elliptical cavity configuration. **c** Axial CT with contrast confirms anterior rotation of the outer shell of the bipolar head with asymmetric wall thickness. Eccentric positioning of the femoral head results in uneven spacing and a distorted central cavity configuration. **d** Sagittal CT with contrast demonstrates a relatively uniform appearance of the outer shell, with decreased evidence of anterior rotation. However, the femoral head remains eccentrically positioned within the outer shell, evidenced by the asymmetric spacing between components. **e** Anterior/posterior radiograph showing bipolar head dissociation following closed reduction. Dashed-ring annotations highlight separation of the outer acetabular shell from the inner femoral head of the component. **f** Radiographic and CT indicators of bipolar cup dissociation risk. Anteroposterior radiograph shows an apparently concentric, radiodense “O” outline of the outer shell; however, subtle asymmetric cortical thickness of the ring suggests anterior rotation. Coronal CT confirms eccentric seating of the inner femoral head within the shell, indicating misalignment despite the deceptive concentric silhouette, a pattern associated with failed or hazardous closed reduction attempts and potential intrareduction dissociation

Sixteen days after the initial right HHA, the patient presented to the emergency department after experiencing a ground-level fall, which resulted in a posterior dislocation of the right hip hemiarthroplasty prosthesis. Prior to any closed reduction attempts, both anteroposterior (AP) radiograph and coronal reformatted computed tomography (CT) scan of the abdomen and pelvis were obtained as part of the trauma evaluation. AP radiograph demonstrated anterior rotation of the prosthesis’ external shell, evidenced by asymmetric radiodensity, despite maintaining an “O”-shaped outline. The internal component also displayed an “O”-shaped border (Fig. 1a). On coronal CT imaging, the external shell outline appeared “O” shaped, although anterior rotation was apparent, indicated by asymmetric border thickness and an elliptical central cavity. The internal component also exhibited an “O”-shaped outline but was eccentrically positioned within the external shell, as indicated by asymmetrical spacing between the internal head and the surrounding shell walls (Fig. 1b). Additional axial and sagittal CT reconstructions demonstrated findings consistent with the coronal images, confirming eccentric positioning of the internal component and anterior rotation of the external shell (Fig. 1c, d). A closed reduction was completed at that time, which resulted in bipolar cup dissociation (Fig. 1e). An open reduction was subsequently performed, with intraoperative findings revealing the external bipolar head component completely dissociated. The bipolar head component was carefully inspected and demonstrated no visible evidence of mechanical damage or polyethylene wear. The femoral stem and neck were well fixed and stable within the femoral canal. A trial bipolar head of identical size was assembled onto the femoral stem, and the hip was reduced, demonstrating excellent stability through 90° of flexion and 60° of internal rotation. Given the intraoperative findings of stable fixation and absence of component damage, a new bipolar head of the same size was implanted onto the femoral stem, secured according to manufacturer specifications and recommendations, and the hip was successfully reduced with stable articulation and no evidence of residual instability or component malposition. The patient did not experience any postoperative complications and was discharged from the hospital 4 days after the procedure. A 1-month follow-up was completed with no recurring dislocation. Radiographs taken at that time showed stable alignment of the right hip hemiarthroplasty revision. The patient denied any recurrent episodes of dislocation at the last follow-up, 1 year post-revision.

## Discussion

Bipolar cup dissociation following hemiarthroplasty remains a relatively rare complication, with most available literature limited to isolated case reports. While a precise incidence rate is difficult to quantify, this complication is markedly uncommon compared to typical dislocation events [5]. Our case adds to this limited literature available, representing one of the few documented instances in which dissociation occurred during attempted closed reduction.

Instances of bipolar component dissociation are reported in two contexts: Early dissociation typically precipitated during attempted closed reduction of a dislocated bipolar hemiarthroplasty, whereas later dissociation has been associated with inner‑bearing issues in specific designs [6, 7]. Consistent with the bottle‑opener mechanism and the report by Sim et al. on the O sign, the current case represents an example of early dissociation [4].

Introduced by Sim et al. the O sign, or O shape, is defined as a concentric circular appearance of the bipolar shell on anteroposterior radiographs and has been suggested to indicate a construct potentially amenable to closed reduction [4]. In this case, radiographs demonstrated an “O”-shaped outline of the external shell, but asymmetric cortical thickness suggested anterior rotation and CT provided additional clarity by confirming eccentric positioning of the inner femoral head within the shell (Fig. 1f). This detailed imaging offered by CT revealed misalignment that was not fully appreciable on radiographs alone, demonstrating how CT can uncover clinically significant deviations that might otherwise be overlooked.

These findings from this case differ from those reported by Sim et al., whose description implies that a concentric “O” shape outline on AP radiograph denotes that a construct is amenable to closed reduction. Our case suggests that the predictive value of the O sign may be contingent on two key factors: (1) symmetry of the external shell’s radiodense rim, which excludes axial rotation or tilt, and (2) central alignment of the inner head within the shell, which can be confirmed with CT. When either criterion is not met, our findings suggest that the utility of the O sign for guiding closed reduction diminishes, and prompt consideration of open reduction strategies may reduce the risk of component disengagement consistent with the bottle-opener mechanism.

By incorporating CT findings, this case supports refining the O sign from a binary marker based solely on radiographic appearance to a more nuanced, composite assessment. CT allows direct evaluation of both shell symmetry and inner head–shell concentricity, revealing misalignments that may not be apparent on radiographs. These insights provide a more accurate appraisal of mechanical alignment and dissociation risk, informing decisions about whether closed reduction is appropriate and potentially reducing the likelihood of intrareduction complications.

In this patient’s arthroplasty, a single-locking Stryker UHR head was used. Prior work suggests single-locking rings may confer higher dissociation risk than dual-locking systems during closed reduction of dislocated bipolar constructs, although data is limited [5]. Notably, dissociation has also been reported with dual-locking designs (for example, the RINGLOC in the O sign report), indicating that mechanism-based vigilance is required regardless of locking type [4]. Consequently, while the locking mechanism type may influence baseline risk, it does not diminish the relevance of this case’s findings to the interpretation and refinement of the O sign, as the radiographic principles and diagnostic implications apply regardless of whether the bipolar head is single or dual locking. However, this report’s findings do align with observations that dissociation frequently occurs during manual reduction and often necessitates operative management, particularly with susceptible locking designs [3, 7].

In conclusion, the findings of this case report are not fully consistent with those described by Sim et al. in their original O sign report. Although Sim et al. originally suggested that a closed reduction could be safely attempted when the O sign was present, this case demonstrates that the additional imaging with CT must be strongly considered before such a reduction is pursued. Consequently, the observations presented here offer a refinement of the O sign, suggesting possible updates to its interpretation and potential clinical utility. While these observations strengthen the conceptual value of the sign, its predictive accuracy remains unproven, as our conclusions are drawn from a single retrospective case. Symmetry of the outer shell’s radiodense rim and central alignment of the inner head—whether seen on radiographs or CT—should not be taken as assurance of successful reduction. Until validated through larger prospective studies, the O sign should be regarded as a useful adjunct rather than a definitive guide, and bipolar dislocation reductions must continue to be performed with careful attention to implant orientation and component integrity.

## Data Availability

Not applicable.
